# P51 A service evaluation into the reduction of blood monitoring frequency during the COVID-19 pandemic

**DOI:** 10.1093/rap/rkac067.051

**Published:** 2022-09-28

**Authors:** Octavio Aragon Cuevas, Arian Webb

**Affiliations:** Alder Hey Children's Hospital NHS Foundation Trust, Liverpool, United Kingdom; Liverpool John Moores University, Liverpool, United Kingdom; Liverpool John Moores University, Liverpool, United Kingdom

## Abstract

**Introduction/Background:**

Due to the immense pressures placed on primary care services and the additional shielding advice for patients on immunosuppressive treatments at the beginning of the COVID-19 pandemic, the rheumatology team at this paediatric tertiary centre decided to decrease the blood monitoring frequency for paediatric rheumatology patients on adalimumab and etanercept monotherapy to 6 monthly from 3 monthly. This service evaluation aims to identify whether this change in monitoring frequency led to a negative impact on patient safety.

**Description/Method:**

A record of all relevant patients was obtained using WellSky® dispensing records. Filters were applied in order to be able to compare data in patients who were on the drugs for sufficient time (6 months) both pre and post change in monitoring frequency (defined as May 2020). Derangements in blood results (neutropenia, thrombocytopenia and liver function tests [LFTs]) were recorded, categorised (clinically significant = neutropenia <1 x 10^9^/L, thrombocytopaenia <100 x 10^9^/L, LFTs >3 fold upper limit of normal [ULN]) and compared. Adherence to suggested blood monitoring frequencies was evaluated pre and post change. It was deemed acceptable by the rheumatology team to be up to 1 month late for blood monitoring

**Discussion/Results:**

585 blood tests were performed across 96 patients between April 2019 and October 2021. There were 101 blood derangements in total (17%, 101/585). 54.5% (55/101) of derangements occurred pre change, with 14.5% (8/55) being classed as clinically significant. Of the derangements that occurred post change, only 6.5% (3/46) were clinically significant and were observed in one patient. All clinically significant derangements (pre and post change) were related to the LFTs. All clinically significant derangements were observed in adalimumab patients. More patients adhered to the monitoring frequency post change (71% post change vs 55% pre change). Patients who had experienced clinically significant derangements pre change, did not show any post change.

**Key learning points/Conclusion:**

This service evaluation has many limitations that impede drawing definite conclusions. For instance, other potential causes of LFT derangements were not recorded. Dose and frequency of administration of adalimumab and etanercept were also not recorded. Nonetheless, changing to 6 monthly monitoring does not appear to have had a negative impact on patient safety. It appears that an increased frequency of blood monitoring in these patients results in an increase detection of mild blood derangements. The incidence of clinically significant derangements in LFTs was reduced post change and clinically significant neutropaenia and thrombocytopaenia were not detected. Lower frequency of monitoring benefits both the NHS, patients and their families. We conclude that for the majority of patients on adalimumab or etanercept monotherapy, the benefits of 6 monthly monitoring outweigh any potential risks and we will continue to apply this approach to our patients in the future.

